# Effect of Dietary Rumen-Degradable Starch to Rumen-Degradable Protein Ratio on In Vitro Rumen Fermentation Characteristics and Microbial Protein Synthesis

**DOI:** 10.3390/ani12192633

**Published:** 2022-09-30

**Authors:** Panliang Chen, Yan Li, Yizhao Shen, Yufeng Cao, Qiufeng Li, Meimei Wang, Mingchao Liu, Zhiyuan Wang, Zihan Huo, Shuai Ren, Yanxia Gao, Jianguo Li

**Affiliations:** 1 College of Animal Science and Technology, Hebei Agricultural University, Baoding 071001, China; 2 Key Laboratory of Healthy Breeding in Dairy Cattle (Co-Construction by Ministry and Province), Ministry of Agriculture and Rural Affairs, Hebei Agricultural University, Baoding 071001, China; 3 College of Veterinary Medicine, Hebei Agricultural University, Baoding 071001, China; 4 Hebei Technology Innovation Center of Cattle and Sheep Embryo, Baoding 071001, China; 5 Hebei Research Institute of Dairy Industry Technology, Shijiazhuang 050221, China

**Keywords:** in vitro, rumen-degradable starch, rumen-degradable protein, rumen fermentation, microbial protein synthesis

## Abstract

**Simple Summary:**

Synchronizing the energy and nitrogen supply in the rumen was reported to improve feed efficiency; however, synchronization indicators were rarely reported. The present study was conducted using a batch culture technique to evaluate the optimal rumen-degradable starch (RDS) and rumen-degradable protein (RDP) ratio (SPR) for synchronizing the energy and nitrogen supply for dairy cows. The in vitro disappearance of dry matter and neutral and acid detergent fiber, gas production and microbial crude protein synthesis quadratically increased with increasing the SPR and peaked at 2.3. Although an in vivo study could be further needed, our results provided an optimal SPR range for dairy cows to synchronize the energy and nitrogen supply.

**Abstract:**

The objective of this study was to investigate the effects of dietary rumen-degradable starch (RDS, g/kg of DM) to rumen-degradable protein (RDP, g/kg of DM) ratios (SPR) on in vitro rumen fermentation characteristics and microbial protein synthesis (MCPS). Treatments were eight diets with SPR of 1.9, 2.0, 2.1, 2.2, 2.3, 2.4, 2.5 and 2.6 and were formulated to be isoenergetic, isonitrogenous, and isostarch. Substrates were anaerobically incubated in sealed culture vials (100 mL) for 6, 24 or 48 h. Three incubation runs were conducted within two consecutive weeks. With the increase of the dietary SPR, the gas production (GP), in vitro dry matter disappearance (IVDMD) and concentration of MCPS and total volatile fatty acids (TVFA) linearly increased after 6 h of incubation (*p* ≤ 0.01), whereas they quadratically increased and peaked at the SPR of 2.3 after 24 and 48 h of incubation (*p* < 0.05). In response to dietary SPR increasing, the in vitro neutral detergent fiber disappearance (IVNDFD) quadratically increased (*p* < 0.01), and the ammonia nitrogen (NH_3_-N) concentration linearly decreased (*p* < 0.01) after 6, 24 and 48 h of incubation. Based on the presented results, an SPR of 2.3 is recommended for formulating a diet due to its greatest IVDMD, IVNDFD, GP, TVFA and MCPS. However, as the results obtained are strictly dependent on the in vitro conditions, further in vivo studies are needed to verify our findings.

## 1. Introduction

The rumen is regarded as a natural anaerobic fermenter inhabited by many microorganisms. There is a strong link between rumen fermentation efficiency and microbial viability [1]. Synchronizing energy and nitrogen (N) supply in the rumen could maximize the growth and activity of rumen microorganisms, thereby potentiating the efficiency of rumen fermentation and microbial protein synthesis (MCPS) [2]. In addition, as microbial protein is the primary source of essential amino acids for dairy cows [3], the increase in MCPS is not only beneficial for optimizing milk protein but also for improving N utilization in dairy cows [4].

Previous experiments have been conducted to explore the effects of a synchronous diet using different indicators, including non-fibrous carbohydrates to degradable intake protein, non-structural carbohydrates to rumen-degradable protein (RDP) and rumen-degradable nitrogen to fermentable organic matter [5,6,7]. However, the results were not consistent, which was likely because the rate of energy or protein availability is often confounded with the total amount of energy or protein availability [8]. Therefore, more attention should be paid to the characteristics of energy and N sources used in diet [9]. Recent studies have demonstrated that rumen-degradable starch (RDS) and RDP could affect rumen fermentation, microbial community compositions, and MCPS [10,11,12,13]. Since MCPS was recognized as the important criterion for determining the degree of dietary synchronization [14,15,16], it is reasonable to infer that the RDS and RDP may be more effective indicators for evaluating the synchrony of energy and N supply. However, the optimal RDS to RDP ratio (SPR) in the diet is unclear.

As little research accurately reported the RDS and RDP contents in the diets of lactating cows, we previously analyzed the chemical compositions of diets for high-production dairy cows from 15 commercial dairy farms and found the value of SPR ranged from 1.9 to 2.6 (unpublished). Therefore, we hypothesized that the SPR could influence microbial activity, thus affecting rumen fermentation and MCPS efficiency. The objective of this study was to evaluate the response of different SPRs to rumen fermentation characteristics and microbial protein synthesis to screen out the optimal SPR.

## 2. Materials and Methods

All animal usage and experimental procedures (JGL 202102) were approved by the Animal Care Committee of Hebei Agriculture University (Baoding, China).

### 2.1. Treatments

Eight experimental diets were formulated according to different SPRs, which were 1.9, 2.0, 2.1, 2.2, 2.3, 2.4, 2.5 and 2.6. The diets contained 31.48% whole-plant corn silage, 12.42% alfalfa hay, 4.94% oat hay and 51.16% concentrate on a dry matter (DM) basis (Table 1). The chemical compositions and effective rumen degradability of the feed ingredients are shown in Table 2. The content of starch, crude protein (CP) and energy were similar among the diets.

### 2.2. Animals and Diet

Three Holstein steers (850 ± 45 kg of BW) equipped with a permanent rumen cannula were used for in situ incubation and served as donor animals for the in vitro experiment. The steers were fed ad libitum a total mixed ration twice daily at 07:00 and 19:00 and were provided free access to water. The diet of the donors contained 40.00% whole-plant corn silage, 20.00% Chinese wild rye, 20.50% ground corn, 4.36% soybean meal, 9.36% cottonseed meal, 3.69% DDGS and 2.09% vitamin and mineral supplement (DM basis), to provide 13.93% of CP, 36.80% of neutral detergent fiber (aNDF) and 28.80% of starch.

### 2.3. In Situ Ruminal Degradation

The effective rumen degradability of the starch (ERDST, Table 2) and protein (ERDCP) of the feed ingredients used in this study were determined using the in situ nylon bags technique [17]. Briefly, the feed ingredients were milled to pass through a 2-mm screen (stand model 4 Wily Mill, Arthur H. Thomas, Philadelphia, PA, USA). The nylon bags (10 cm × 20 cm with a 50 μm pore size) were pre-weighed and filled with either 7.0 g of concentrate or 4.0 g of forage (DM basis). Prior to incubation, the bags were soaked for 10 min in warm tap water (39 °C). Four replicates per time point were incubated in each steer. Bags with concentrate were taken out after 2, 4, 8, 16, 24 and 48 h of incubation, and bags with forage were taken out after 4, 8, 16, 24, 48 and 72 h of incubation. After each incubation time point, bags were removed from the rumen and immediately dispersed in ice water to stop further degradation and rinsed with cold tap water to remove rumen digestive residues. The rinsed nylon bags were dried in a forced-air oven at 55 °C for 48 h and weighed. Residues were stored at 4 °C for chemical analysis. The degradation parameters and effective ruminal degradability (ERD) were calculated according to Ørskov and McDonald [18]:

(1) $$Y_{t}=a+b\times \left(1-e^{-\mathrm{kt}}\right)$$

(2) $$\mathrm{ERD}=a+b\times k/\left(k+k_{p}\right)$$

where Y_t_ = disappearance proportion at time t; a = rapidly degradable fraction; b = slowly degradable fraction; k = constant rate of degradation of fraction b; t = time of incubation (h); k_p_ = passage rate, the rumen outflow rate was set at 0.06/h according to Offner et al. [19].

Dietary RDS or RDP was calculated using the following equation [20]:

(3) $$\mathrm{RDS}\;(\mathrm{RDP})=\sum_{i=1}^{n}P_{i}\times {\mathrm{ERD}}_{i}$$

where P_i_ represents the proportion of dietary starch or protein of feed i in the diet, ERD_i_ represents the effective starch or protein rumen degradability of feed i, and n is the number of ingredients containing starch or protein in the diet.

### 2.4. Batch Culture Procedure

Prior to in vitro incubation, 500 ± 0.5 mg (DM basis) of the substrate was weighed into an acetone-washed and pre-weighed Ankom filter bag (F57; Ankom Technology, Macedon, NY, USA), then bags were heat sealed and placed into 100 mL culture vials. Blank with a pre-treated filter bag was incubated without any substrate to correct the gas production (GP). Fresh rumen fluid was collected from the donor steers before morning feeding, squeezed through four-layer cheesecloth, transferred in a pre-warmed bottle at 39 °C after pH measurement, and immediately transferred to the laboratory, then kept at 39 °C water bath and bubbled with CO_2_. The time from rumen-fluid collection to inoculation was no more than 30 min. Fresh buffer was prepared according to Menke et al. [21], kept at 39 °C in a water bath, and bubbled with CO_2_. A total of 45 mL buffer and 15 mL of rumen fluid were added to each vial and bubbled with CO_2_ for 15 s to maintain anaerobic conditions. Then, the vials were immediately sealed with butyl rubber stoppers and aluminum crimp seals. Thereafter, all the vials were incubated at 39 °C and oscillated at 125 rpm in a gas bath shaker (GCTS-2018, Jingda, Inc., Jintan, China). The pH of the buffer and rumen fluid mixture was averaged at 7.0 ± 0.21. Both blanks and treatments had three replicates in each sampling time point (6, 24, and 48 h). Three batch culture runs were performed within two weeks.

### 2.5. Gas Production and Sampling

Headspace gas pressure was recorded at 3, 6, 9, 12, 24, 36 and 48 h using the pressure meter as described by Theodorou et al. [22]. The gas was then released by leaving the needle in the vial after pressure measurement. The cumulative GP was calculated according to Mauricio et al. [23]:

GP_t_ (mL) = 0.18 + 3.697P_t_ + 0.0824P_t_^2^ (4)

where GP_t_ is the GP volume at time ‘t’ (h), P_t_ is the gas pressure measured at the time ‘t’ (h). Gas production kinetics was calculated according to Ørskov and McDonald [18]:

y = GV × (1 − e^− C × [t − lag]^) (5)

where ‘y’ is the cumulative volume of gas produced after ‘t’ hours, ‘GV’ is the asymptotic gas volume, ‘C’ is the rate constant of GP and ‘lag’ is the time (h) between inoculation and commencement of GP. The parameters ‘GV’ and ‘C’ were used to calculate the absolute initial GP during the first hour (Abs):

Abs (mL/g DM) = GV × (1 − e^− C^) (6)

At the end of each incubation time (6, 24 and 48 h), the vials were put on ice to stop fermentation, and the pH of the fermentation liquid was immediately determined. Approximately 20 mL fermentation liquid of each bottle was sampled, mixed with 1 mL 0.25 (wt/vol) HPO_3,_ and kept at −20 °C for volatile fatty acids (VFAs) analysis. Approximately 5 mL fermentation liquid was sampled for MCPS determination, and another 5 mL liquid sample was mixed with 1 mL 0.01 (vol/vol) H_2_SO_4_ solution for ammoniacal nitrogen (NH_3_-N) determination. The Ankom filter bags were washed with cold water and dried at 55 °C for 48 h to determine the undigested substrate CP concentration, the in vitro dry matter disappearance (IVDMD), neutral detergent fiber disappearance (IVNDFD), acid detergent fiber disappearance (IVADFD), and hemicellulose disappearance (IVHCD).

### 2.6. Chemical Analysis

The DM (method 930.15), ether extract (method 991.36), CP (method 968.06), starch (method 996.11) and ash (method 942.05) were determined following the methods of AOAC (2005) [24]. The aNDF, analyzed using a heat-stable amylase, and ADF were expressed as the inclusion of residual ash according to the method described by Van Soest et al. [25]. The IVDMD, IVNDFD, IVADFD and IVHCD were calculated as the weight difference of DM, aNDF, ADF and HC in the substrate before and after incubation. The concentration of NH_3_-N and VFAs were analyzed as described by Broderick and Kang [26] and Erwin et al. [27], respectively. The MCPS was determined as described by Makkar et al. [28].

### 2.7. Statistical Analysis

All data were analyzed using the PROC MIXED model procedure of SAS (SAS Inst. Inc., Cary, NC, USA) with a model including SPR as a fixed effect and the incubation run as a random effect. Significant differences between the treatment means were identified using the least significant means. Polynomial contrasts were used to determine linear (L) and quadratic (Q) responses to the SPR. Significant effects were declared at *p* ≤ 0.05.

## 3. Results

### 3.1. In Vitro Nutrients Disappearance and Gas Production

**Figure 1:**
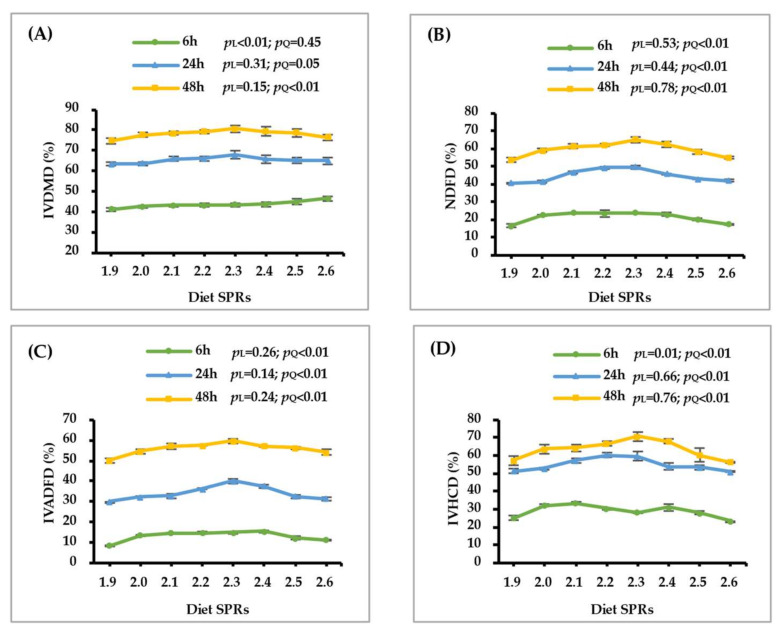
Effect of dietary rumen-degradable starch to rumen-degradable protein ratio (SPR) on in vitro dry matte disappearance (IVDMD, (**A**), in vitro neutral detergent fiber disappearance (IVNDFD, (**B**)), in vitro acid detergent fiber disappearance (IVADFD, (**C**), and in vitro hemicellulose disappearance (IVHCD, (**D**)) after 6, 24, and 48 h of incubation. Error bars indicate measure of variation within the dietary SPRs. L is linear, and Q is quadratic effects for diet SPR.

As shown in Figure 1 and Table 3, with the increase of dietary SPR, the IVDMD and GP linearly increased after 6 h (*p* ≤ 0.01) of incubation and quadratically increased after 24 h (*p* ≤ 0.05) and 48 h (*p* < 0.01) of incubation. Increasing dietary SPR caused a quadratic increase in the IVNDFD, IVADFD and IVHCD after 6, 24 and 48 h of incubation (*p* < 0.01). Additionally, the GV, C, Lag time and Abs quadratically increased with the dietary SPR increasing (*p* ≤ 0.01).

### 3.2. Rumen Ammonia Nitrogen Concentration and Microbial Protein Synthesis

As shown in Table 4, a linear reduction in NH_3_-N concentration was detected after 6, 24 and 48 h of incubation (*p* < 0.01) with the increase of dietary SPR, whereas a linear increase was observed in undegraded CP content after 6 and 48 h of incubation (*p* < 0.01). When expressed as mg N/mmol of VFA, the MCPS concentration was not affected by treatments, except for a quadratic increase after 24 h of incubation (*p* = 0.02). However, when expressed as mg N/g DM of the incubated substrate, the MCPS linearly increased with the increasing dietary SPR after 6 h of incubation (*p* < 0.01) and quadratically after 24 and 48 h of incubation (*p* < 0.01).

### 3.3. Rumen Fermentation Characteristics

As shown in Table 5, although the rumen pH was not affected by the dietary SPR, the Total VFA (TVFA) concentration showed a linear increase after 6 h of incubation (*p* < 0.01) and a quadratic increase after 24 h (*p* = 0.02) and 48 h (*p* < 0.01) of incubation with the increasing dietary SPR. Furthermore, after 24 h of incubation, the molar proportion of butyrate and acetate to propionate ratio (A:P) quadratically increased (*p* ≤ 0.02), and the molar proportion of valerate and branched-chain VFA (BCVFA, including isobutyrate and isovalerate) linearly increased (*p* < 0.01), whereas, the molar proportion of propionate quadratically decreased with the increasing dietary SPR (*p* < 0.01). In addition, after 48 h of incubation, increasing dietary SPR linearly increased the A:P ratio and the molar proportions of acetate and isovalerate (*p* ≤ 0.02), and quadratically increased the molar proportion of butyrate (*p* = 0.03), while it linearly decreased the molar proportion of propionate (*p* < 0.01).

## 4. Discussion

### 4.1. In Vitro Nutrients Disappearance and Gas Production

The IVDMD after 6 h of incubation has generally been considered as the loss of the rapid degradation fraction of the substrate [29]. Since starch could be the dominant rapid degradation fraction in the present study [30], the IVDMD after 6 h of incubation could be mainly affected by the RDS content in the diet. Wheat has a higher RDS content than corn due to its lower crystallinity of starch granule and looser starch–protein linkage in the endosperm [31]. In this study, the higher SPR diet had a higher proportion of wheat; thus, the IVDMD after 6 h of incubation increased with the increasing dietary SPR. Whereas, after 24 h and 48 h of incubation, the IVDMD was more related to rumen microbial activity. Under adequate N source supply conditions, evaluating the supply of carbohydrates could increase the availability of ATP and carbon skeleton, thus improving microbial growth efficiency and increasing the digestibility of nutrients [32]. The results of this study corroborate the above findings; the IVDMD elevated as increasing dietary SPR from 1.9 to 2.3. When dietary SPR was greater than 2.3, the higher proportion of rumen-protected soybean in the diet induced a lower dietary RDP content. The deficiency in nitrogen supply could limit microbial growth [12], consequently leading to decreased IVDMD.

As we know, a diet of high fermentable carbohydrates can depress fiber degradation by decreasing the ruminal pH [33]. However, in the current study, there was no change in pH value among diets with different SPR. As previously reported by Lechartier and Peyraud [34], the high content of highly degradable starch in the diet could impair fibrolytic activity independently of its effect on ruminal pH. The evaluated dietary RDS content may increase the competition between amylolytic bacteria and cellulolytic bacteria in the rumen. Ren et al. [10] also reported that the relative abundance of amylolytic bacteria was increased, whereas the cellulolytic bacteria were decreased in the heifers fed a high RDS diet compared with a low RDS diet. Moreover, cellulolytic bacteria are also sensitive to a shortage of N [2]. When dietary SPR was at a high level, the higher RDS content and lower RDP may depress the growth of cellulolytic bacteria, consequently leading to lower fiber degradation. Therefore, the quadratic response of the increasing dietary SPR to the fiber degradation in the present study could be acceptable.

Gas is always released along with the microbial degradation of the substrate, and in a batch culture system, the GP was reported to be strongly related to IVDMD [35]. Thus, the variations in GP and GP kinetics in this study were mainly caused by the change in IVDMD. With the increase of the dietary SPR, the GP linearly increased after 6 h of incubation, which indicated the initial rate of digestion was enhanced, whereas the GP rate and GP after 24 h and 48 h of incubation quadratically increased, which reflected the change in the extent of diet digestion on the whole incubation [29,36].

### 4.2. Rumen Ammonia Nitrogen Concentration and Microbial Protein Synthesis

In ruminants, rumen concentration of NH_3_-N could be an important indicator to reflect rumen fermentation. The rumen NH_3_-N is mainly produced by CP degradation of the substrate and eliminated by outflowing of rumen content, absorption through the rumen wall and the use of microbial. As the main nitrogen source for rumen microbes [37], NH_3_-N concentration was commonly recognized as an indicator reflecting the synthesis ability of microbial protein in vivo, and the lower NH_3_-N concentration usually indicates that more NH_3_-N is used for MCPS [38]. However, in the present study, there was no rumen content outflow or rumen wall absorption in the batch culture system; thus, the rumen NH_3_-N concentration could only be affected by feed degradation and microbial use [39]. In the current study, since the linearly decreased NH_3_-N concentration was in consistent with the linearly increased undegradable CP, not the quadratically increased MCPS, the changes in NH_3_-N concentration among treatments could mainly be affected by different RDP content in different diets.

In addition, the NH_3_-N concentration of diets in the present study were all more than 5 mg/dL. That seems to suggest that each diet could provide sufficient N for MCPS [40]. However, Schwab et al. [41] suggested that the optimum concentration of ruminal NH_3_-N seemed to be diet-dependent and influenced by carbohydrate fermentability, and a greater ruminal NH_3_-N concentration may be required if more rapidly fermentable carbohydrates are supplied. Odle and Schaefer [42] reported that greater ruminal NH_3_-N concentrations were required for ground barley (8.9 mM) than for ground maize (4.3 mM) to maximize in situ digestion rate. In the present study, due to the increased RDS content with increasing dietary SPR, the demand for NH_3_-N and the ability to capture N by rumen microbes may increase. The high SPR diet may need more rumen-degradable N and greater minimum NH_3_-N concentration thresholds. Therefore, although the NH_3_-N concentration is greater than 5 mg/dL, it does not mean that the requirement of N for maximizing MCPS can be satisfied.

Microbial protein is dairy cows’ primary source of essential amino acids [3]. Synchronizing energy and nitrogen supply could evaluate MCPS efficiency via potentiate enzyme activities of ammonia assimilation and increase the abundance of the rumen microbes of nitrogen metabolism [43]. MCPS was recognized as the important criterion for determining if a treatment is more synchronous or not [14,15,16]. In the current study, the MCPS (mg N/g DM) increased with increasing dietary SPR after 6 h incubation. That result suggested that, in the initial stage of fermentation, energy supplementation can enhance the degrees of synchroneity of diet. However, after 24 h incubation, the MCPS (mg N/g DM) and MCPS (mg N/mmol TVFA) decreased when dietary SPR was greater than 2.3. That might be caused by the asynchronous supply of energy and nitrogen. As the dietary SPR exceeded 2.3, the degraded rate of proteins might be slower than the degraded rate of carbohydrates; thus, the insufficient supply of N made the production of ATP unavailable for MCPS [9].

Moreover, increasing the abundance of carbohydrate species contributes to elevating the diversity of rumen microorganisms, thus increasing the MPCS [2]. In the present study, the differences in the content of beet pellets among diets may be a factor causing the change in MCPS. However, as reported by Münnich et al. [44], no significant changes in most rumen microorganisms were observed when the dietary beet pellets content was increased from 8.00% to 16.00%. Voelker and Allen [45] reported no change in MCPS even with the application of beet pellets at 24% of the diet. In the present study, though the content of beet pellets increased with the increase of dietary SPR, the content of beet pellets ranged from 9.26 to 12.64%, which were all in the range of 8.00 to 16.00%. Thus, the difference in the content of beet pellets was not a major influencing factor for MCPS.

Furthermore, the undegraded CP increased with declining RDP proportion suggesting that lowering dietary RDP could conserve protein and make it more available for the small intestine [46]. Combined with the results of MCPS, the appropriate dietary SPR may increase the supply of metabolic proteins for the dairy cow, and the ratio of 2.3 may achieve the balance.

### 4.3. Rumen Fermentation Characteristics

Rumen pH is a key factor in the regulation of microbial activity. Due to higher acid production for high RDS content and lower bicarbonates production for low RDP content [33,34], a high SPR diet was expected to diminish ruminal buffering capacity and then reduce ruminal pH. However, for the batch culture technique used in the present study, the buffer should be sufficiently added at the beginning, and thus, the pH should be less sensitive to TVFA production [47]. Thus, the TVFA concentration significantly changed in the fermentation liquid, while no differences observed in pH should be acceptable, and similar results were also previously reported by Shen et al. [48].

The TVFA concentration followed a similar response with the IVDMD and MCPS. That may contribute to the variation of rumen microorganisms’ abundance, which led to the change in IVDMD, consequently influencing TVFA production. Regarding VFAs, after 24 h of incubation, the quadratic increase for the A:P ratio by increasing dietary SPR was caused by the change of proportion of propionate. And after 48 h of incubation, as dietary SPR increased, the increment in the proportion of acetate and reduction in the proportion of propionate resulted in the linear increase in the A:P ratio. A similar result was observed by Kand et al. [49], who also found that acetate proportion was higher and propionate proportion was lower in corn starch diets than in cellulose diets by comparing three carbohydrate sources (sucrose, corn starch and cellulose). One possible explanation was that, as incubation time increased, the rapidly degradable carbohydrates (i.e., RDS) decreased, whereas the slow-degradable carbohydrates increased in the substrate [50]. Moreover, as fibrous carbohydrates are a major precursor of butyrate [51], the quadratically increased molar proportion of butyrate with the increase of SPR after 24 and 48 h of incubation may be caused by the variation of fibers degradation in this study. Additionally, the increase in valerate proportions is more associated with the proportion of ground wheat in the diet. Wheat is rich in proline [52], which is a precursor for valerate; thus, a diet with more wheat could produce more valerate.

BCVFAs are derived from the deamination and decarboxylation of branched-chain AA, such as valine, leucine, and isoleucine [53]. A reduction in protein degradation in the rumen has been associated with a decrease in the concentration of BCVFA [54,55]. However, in the present study, the molar proportion of BCVFA increased with the dietary RDP content decreased. Calsamiglia et al. [56] found that the flow of the branched-chain AA from a continuous culture fermenter was higher with lignosulfonate-treated soybean meal than with untreated soybean meal. Kand et al. [54] suggested that, in response to a lack of N, the predation and autolysis of microbes might increase to support more amino acids for BCVFA production. In this experiment, at a high SPR diet, the increase in BCVFA from the breakdown of microbial protein was presumably greater than the decrease in BCVFA from the degradation of dietary protein, leading to an overall increment in BCFA proportions. In addition, the amount of rumen-protected soybean meal increased with the dietary SPR increased, which could also be an important consideration for the increase in BCVFA.

## 5. Conclusions

The changes in rumen fermentation characteristics and MCPS suggested that the SPR could affect rumen microbial activity. According to the greater IVDMD, IVNDFD, GP, TVFA and MCPS in the present study, an SPR of 2.3 could be an optimal ratio for dairy cows. However, further research on rumen microbial composition is expected to get a clearer explanation for those changes. As our results are restricted to in vitro conditions, in vivo studies are needed to investigate the practical benefits to animal performances.

## Figures and Tables

**Table 1:** Ingredients and chemical compositions of diets with different rumen-degradable starch to rumen-degradable protein ratio (SPR).

Item ^1^	Treatments (SPRs)							
	1.9	2.0	2.1	2.2	2.3	2.4	2.5	2.6
Ingredients (% DM)								
Whole-plant corn silage	31.48	31.48	31.48	31.48	31.48	31.48	31.48	31.48
Alfalfa hay	12.42	12.42	12.42	12.42	12.42	12.42	12.42	12.42
Oat hay	4.94	4.94	4.94	4.94	4.94	4.94	4.94	4.94
Ground corn	19.76	17.71	13.77	11.80	7.87	5.90	3.93	0.00
Ground wheat	0.00	2.07	6.00	7.97	11.90	13.87	15.83	19.77
Soybean meal	13.96	12.79	9.84	7.38	5.90	4.13	1.97	0.00
Rumen-protected soybean meal	0.00	0.98	2.95	4.92	5.71	6.89	8.85	10.33
Wheat bran	2.79	2.94	2.95	2.95	2.95	2.95	2.95	2.95
Beet pellets	9.26	9.28	10.26	10.75	11.44	12.03	12.16	12.64
Fat powder	2.36	2.36	2.36	2.36	2.36	2.36	2.44	2.44
Premix ^2^	3.03	3.03	3.03	3.03	3.03	3.03	3.03	3.03
Chemical compositions (% DM)								
CP	15.75	15.83	15.71	15.66	15.65	15.56	15.61	15.68
EE	4.66	4.69	4.75	4.78	4.83	4.86	4.97	5.03
Ash	7.39	7.40	7.42	7.43	7.45	7.46	7.47	7.48
Starch	26.63	26.63	26.62	26.62	26.59	26.59	26.56	26.53
aNDF	32.95	33.18	33.84	34.25	34.73	35.11	35.38	35.84
ADF	17.18	17.21	17.41	17.52	17.67	17.79	17.82	17.94
HC	15.77	15.97	16.43	16.73	17.06	17.32	17.56	17.90
RDS	18.55	18.99	19.85	20.27	21.12	21.55	21.95	22.78
RDP	9.54	9.51	9.31	9.11	9.10	8.96	8.80	8.75
SPR	1.94	2.00	2.13	2.23	2.32	2.41	2.49	2.60
NFC ^3^	39.25	38.90	38.27	37.88	37.33	37.01	36.58	35.97
NE_L_ (Mcal/kg of DM)	1.70	1.70	1.69	1.69	1.68	1.67	1.67	1.67

^1^ DM = dry matter; CP, crude protein; EE, ether extract; aNDF, neutral detergent fiber determined using heat-stable amylase and expressed inclusive of residual ash; ADF, acid detergent fiber expressed inclusive of residual ash; HC, hemicellulose; RDS, rumen degradable starch; RDP, rumen degradable protein; SPR, RDS to RDP ratio; NE_L_, net energy for lactation. ^2^ Premix contained salt, minerals, and vitamins. ^3^ NFC: non-fibrous carbohydrate; NFC% = 100% − CP% − EE% − NDF% − Ash%.

**Table 2:** Effective ruminal degradability and content for starch and crude protein (CP) of principal dietary ingredients.

Item ^1^	Whole-Plant Corn Silage	Oat Hay	Alfalfa Hay	Ground Wheat	Ground Corn	Soybean Meal	Rumen- Protected Soybean Meal	Wheat Bran	Beet Pellets
Chemical compositions (% DM)									
Starch	34.05	6.67	3.76	63.90	65.62	3.50	3.52	22.60	9.62
RDS	27.80	4.32	2.26	60.19	39.55	2.28	1.65	14.85	8.14
CP	8.50	7.30	19.36	15.54	9.16	49.16	50.68	19.50	11.19
RDP	5.77	3.62	12.04	10.52	6.38	25.77	15.85	14.86	9.14
ERD (%)									
ERDST	81.65	64.81	60.00	94.19	60.27	65.12	46.86	65.70	84.57
ERDCP	67.92	49.64	62.17	67.06	58.08	54.42	31.27	76.18	81.72

^1^ DM = dry matter; RDS, rumen degradable starch; RDP, rumen degradable protein; ERD, effective ruminal degradability; ERDST, effective ruminal degradability for starch; ERDCP, effective ruminal degradability for crude protein.

**Table 3:** Effects of dietary rumen-degradable starch to rumen-degradable protein ratio (SPR) on in vitro gas production parameters.

Items ^1^	Treatments (SPRs)								SEM ^2^	*p*-Value ^3^	
	1.9	2.0	2.1	2.2	2.3	2.4	2.5	2.6		L	Q
GP, mL/g DM											
6 h	18.4	20.7	21.0	21.7	22.6	23.9	24.1	24.2	0.78	<0.01	0.10
24 h	83.2	86.8	94.5	99.7	102.9	96.3	95.8	92.8	3.17	0.01	<0.01
48 h	117	122	130	136	139	137	133	129	3.6	<0.01	<0.01
GP kinetics after 48 h of incubation											
GV, mL/g DM	114	119	126	134	135	136	130	126	3.4	<0.01	<0.01
C,/h	4.65	4.81	5.45	5.73	5.93	4.98	5.24	5.00	0.272	0.33	<0.01
Lag, h	2.04	1.85	2.18	2.21	2.13	1.19	1.43	1.25	0.167	<0.01	0.01
Abs, mL/g DM	5.22	5.59	6.66	7.36	7.79	6.59	6.63	6.15	0.421	0.04	<0.01

^1^ GP = gas production; DM = dry matter; GV = asymptotic cumulative gas volume; C = rate of fermentation; Lag = lag time; Abs = absolute initial gas production during the first hour. ^2^ SEM, standard error of means. ^3^ L = linear; Q = quadratic.

**Table 4:** Effects of dietary rumen-degradable starch to rumen-degradable protein ratio (SPR) on the concentration of ammonia nitrogen (NH_3_-N), microbial protein synthesis (MCPS), and undegraded crude protein (CP) at different in vitro incubation times.

Items ^1^	Treatments (SPRs)								SEM ^2^	*p*-Value ^3^	
	1.9	2.0	2.1	2.2	2.3	2.4	2.5	2.6		L	Q
NH_3_-N (mg/dL)											
6 h	6.73	6.54	6.46	6.13	5.96	5.71	5.61	5.49	0.111	<0.01	0.62
24 h	7.58	7.31	7.03	6.66	6.39	6.32	6.28	6.06	0.151	<0.01	0.07
48 h	11.14	10.60	10.37	10.15	9.89	9.55	9.30	8.98	0.293	<0.01	0.83
MCPS (mg N/g DM of incubated substrate)											
6 h	2.54	2.57	2.61	2.74	3.22	3.24	3.24	3.00	0.101	<0.01	0.06
24 h	6.90	7.19	7.62	7.81	8.07	7.32	7.23	6.89	0.187	0.85	<0.01
48 h	8.58	8.99	9.43	9.52	10.33	9.93	9.75	9.48	0.260	0.01	<0.01
MCPS (mg N/mmol of TVFA)											
6 h	0.28	0.28	0.28	0.28	0.31	0.31	0.31	0.30	0.014	0.09	0.88
24 h	0.51	0.52	0.52	0.53	0.54	0.50	0.49	0.48	0.016	0.02	0.02
48 h	0.60	0.59	0.58	0.59	0.61	0.62	0.61	0.62	0.024	0.29	0.69
Undegraded CP (g/100 g of CP)											
6 h	58.9	59.5	60.0	59.6	61.0	63.6	63.5	62.6	0.78	<0.01	0.99
24 h	38.7	39.6	40.5	40.7	40.8	41.2	41.7	41.4	1.68	0.16	0.62
48 h	11.2	14.7	15.2	14.5	14.7	17.6	18.7	18.6	1.87	<0.01	0.90

^1^ DM = dry matter; TVFA = total volatile fatty acid. ^2^ SEM, standard error of means. ^3^ L = linear; Q = quadratic.

**Table 5:** Effects of dietary rumen-degradable starch to rumen-degradable protein ratio (SPR) on in vitro rumen fermentation parameters after 6, 24, and 48 h of incubation.

Items ^1^	Treatments (SPRs)								SEM ^2^	*p*-Value ^3^	
	1.9	2.0	2.1	2.2	2.3	2.4	2.5	2.6		L	Q
pH											
6 h	6.72	6.72	6.71	6.71	6.71	6.70	6.71	6.71	0.006	0.20	0.29
24 h	6.61	6.60	6.58	6.58	6.60	6.59	6.60	6.58	0.007	0.14	0.30
48 h	6.60	6.57	6.57	6.58	6.59	6.59	6.57	6.60	0.024	0.83	0.44
TVFA, mmol/L											
6 h	72.7	76.4	79.5	83.8	86.4	86.8	88.3	82.8	2.69	<0.01	0.01
24 h	113	115	122	123	126	123	122	121	3.4	0.03	0.02
48 h	119	126	135	136	140	135	133	128	3.2	0.03	<0.01
Acetate, %											
6 h	65.1	64.5	64.5	64.8	64.1	63.9	63.6	63.8	0.86	0.18	0.93
24 h	62.4	62.4	62.6	62.2	61.7	62.0	62.2	61.6	0.70	0.29	0.94
48 h	58.2	58.3	58.3	59.5	58.7	59.6	58.3	60.2	0.52	0.02	0.99
Propionate, %											
6 h	21.3	21.4	21.3	21.4	21.8	21.4	22.0	21.9	0.44	0.17	0.75
24 h	22.3	22.1	21.2	20.9	20.7	21.2	21.4	21.6	0.28	0.04	<0.01
48 h	23.5	22.7	22.4	22.7	22.0	22.2	21.3	21.6	0.32	<0.01	0.54
Butyrate, %											
6 h	9.7	10.3	10.3	10.0	10.2	10.8	10.7	10.4	0.45	0.12	0.51
24 h	11.9	13.0	12.6	13.1	13.6	13.0	12.7	12.8	0.30	0.09	<0.01
48 h	12.9	13.2	13.7	12.7	13.5	12.4	14.3	12.6	0.13	0.97	0.03
Valerate, %											
6 h	1.75	1.75	1.73	1.69	1.72	1.71	1.66	1.74	0.063	0.50	0.60
24 h	1.45	1.61	1.51	1.53	1.65	1.62	1.65	1.67	0.049	<0.01	0.74
48 h	2.34	2.27	2.10	2.06	2.20	2.42	2.37	2.43	0.089	0.07	0.01
Isobutyrate, %											
6 h	0.66	0.64	0.68	0.66	0.69	0.71	0.65	0.60	0.060	0.72	0.37
24 h	0.61	0.61	0.62	0.70	0.73	0.79	0.62	0.83	0.035	<0.01	1.00
48 h	1.06	1.43	1.28	1.02	1.25	1.30	1.29	1.11	0.087	0.95	0.33
Isovalerate, %											
6 h	1.50	1.50	1.48	1.44	1.47	1.46	1.42	1.48	0.054	0.47	0.59
24 h	1.36	1.63	1.39	1.48	1.58	1.42	1.42	1.53	0.034	0.46	0.34
48 h	2.02	2.12	2.23	2.03	2.33	2.01	2.46	2.14	0.038	<0.01	0.11
BCVFA, %											
6 h	2.16	2.14	2.16	2.10	2.16	2.17	2.07	2.09	0.087	0.49	0.78
24 h	1.97	2.24	2.01	2.18	2.31	2.20	2.04	2.37	0.050	<0.01	0.51
48 h	3.08	3.55	3.51	3.05	3.58	3.31	3.75	3.25	0.121	0.20	0.23
A:P											
6 h	3.06	3.02	3.03	3.04	2.94	2.99	2.89	2.91	0.098	0.16	0.83
24 h	2.81	2.83	2.95	2.97	2.97	2.93	2.91	2.85	0.062	0.45	0.02
48 h	2.48	2.57	2.61	2.63	2.67	2.69	2.74	2.79	0.059	<0.01	0.82

^1^ TVFA = total volatile fatty acid; BCVFA = branched-chain volatile fatty acid; A:P = acetate to propionate ratio. ^2^ SEM, standard error of means. ^3^ L = linear; Q = quadratic.

## Data Availability

The data presented in this study are available on request from the corresponding author.
